# The Nursing Supplement

**Published:** 1888-06-02

**Authors:** 


### The Nursing Mirror.

All Contributions for this Supplement should be addressed "The Nursing Mirror," care of The Editor, 38, Old Jewry, E.C.

### En Passant.

A FEMALE HOUSE SURGEON.—A young American lady, Miss Klumkee, who came to Paris from San Francisco some years ago to study medicine, was the first lady house-surgeon in France. Miss Klumkee is shortly to be married to Dr. Dejerine, and will then undertake a private practice amongst her own sex: her partnership with Dr. Dejérine being both professional and matrimonial.

A PATIENT'S ESCAPADE.—Thomas Crossland, an inmate of the Sheffield Infirmary, had a desperate struggle with two nurses last week. Clad in his night shirt, he ran to a top storey window of the institution. Two nurses grappled with him. Releasing himself from their grasp, Crossland jumped through the window to the ground beneath, where he was picked up fearfully injured. He is not expected to recover.

A WOMAN'S WORK.—All Mr. Besant's heroines are thrown into the shade by the exploits of Miss Beatrice Potter. This young lady is the daughter of Mr. Potter, at one time member for Rochester, and sister of Mrs. Leonard Courtney. Miss Beatrice Potter, when her sister married, carried on the work of rent collecting in Whitechapel, which Mrs. Courtney had commenced. Being greatly struck with the trials of needlewomen, Miss Potter joined their ranks for a time that she might the more fully sympathise with their needs. For three weeks she worked twelve hours a-day in four different East-end sweating dens, and she has lately detailed her experiences before the House of Lords Committee on the Sweating System. The practical information given by Miss Potter may be of the greatest use to the poor needlewomen suffering from this system; but with a splendid sense of honour Miss Potter refuses to betray the names of the shops where she collected her facts.

THE STRIKE AT SHEFFIELD.—A letter in the *Sheffield Daily Telegraph* explains the action taken by the Lady Superintendent of the Nurses' Home in that town. It appears that eleven years ago, when Miss Corvan first accepted the post, there were only 16 nurses, and the institution was in debt; now there are 49 nurses and a surplus at the bankers, yet the committee refuse to make the necessary additions to the home. For 49 nurses there are only two bed-rooms, containing altogether nine beds, and there is only one sitting-room for meals, rest, recreation, etc. How the nurses have submitted so long to such an unhealthy and uncomfortable state of affairs is the only wonder, and it is perfectly natural they should uphold Miss Corvan in her efforts to secure them quiet and comfort during their hours of sleep. The lady superintendent herself was also compelled to give up her room one day every week, and write her letters and conduct her business in the kitchen or passages as she best could. Considering how greatly the work of the institution had increased in Miss Corvan's hands, and how flourishing were its funds, it seems almost incredible that the committee could have been so blind as to refuse to enlarge the home. The Mayor of Sheffield is reported to have said that he should regard Miss Corvan as dead when her notice expired; but perhaps he will find this a difficult feat if Miss Corvan decides to give way to the solicitations of her nurses and the Sheffield people, and carry on her valuable work in that town without the aid of a committee.

ODE TO LABOUR.—We wonder if nurses are familiar with the following beautiful lines in their praise, written by Lewis Morris:—

"Or shall I silence keep
Of you, oh ministering women fair,
Who, while the world lies sunk in careless sleep,
Still for the love of God and man can bear
To watch by alien sick-beds, and to guard
With little hope and scant reward,
Midst misery and foul, infected air,
The friendless and the dying? Shall I dare
To sing of labour's meed nor hold you dear?
Dear souls, your joys are great, and yet not wholly here;
In heaven they blossom best and grow complete,
And beautiful upon the eternal mountains are your feet."

NURSING AS A PROFESSION.—Miss E. A. Barnett, who has been giving some lectures on nursing at Highgate under the auspices of the National Health Society, has proved herself a prudent and suitable teacher. She warned her audience that many small ailments and little defects of character and training, which may pass unnoticed in ordinary life, are serious drawbacks to a nurse's career. Much of a nurse's duty, she explained, consists in doing housemaid's and lady's-maid's work deftly and well, and she advised would-be nurses to begin by learning at home how to make a bed, sweep a room, wash a child, cook a simple meal, and clean ordinary household utensils. If to this were added a little knowledge of elementary physiology and enough Latin to understand ordinary medical terms, they would find their career as a probationer simpler and more profitable. And we may add that if aspirants would try to follow Miss Barnett's instructions at home, they would soon find out whether or not they were capable of the work, and many would thus refrain from entering the ranks of an already crowded profession.

NURSES IN PHILADELPHIA.—The Philadelphia Hospital has 1,600 beds, divided into eleven departments, such as men's medical, maternity, &c.; and each of these departments is under a charge nurse, who is responsible to the chief nurse or matron. Pupils entering the training school for two years receive a salary equivalent to a guinea a month; those entering for a year receive no salary, but are provided with board, lodging, and washing. There is a preliminary examination in reading, writing, and arithmetic for all nursing candidates. The nurses are carefully instructed in ward work, preparation of food for the sick, methods of observation, anatomy, and physiology, during their first year, and on passing an examination are presented with a diploma. Those who pass well and desire to fit themselves for superior positions, are then further taught hospital housekeeping, correspondence, etc. Every nurse has to provide herself with uniform, books, and thermometer, and her expenses for the first year are estimated at a hundred dollars. The rules for nurses and the hours of work are much like those prevalent in London hospitals. Day nurses rise at six and come on duty at seven. They have half-an-hour for dinner in the middle of the day and two hours for recreation in the afternoon. At five they have a meal called "supper," a pretty substantial meal; and at half-past eight they leave the wards. Ten o'clock is bed time. These hours are moderate, and the arrangements for teaching the nurses seem excellent. It is strange that with such a school there should so constantly be complaints of American private nurses.

### Appointments.

Miss Gilpin, sister of the "Lewis Loyd" ward at St. Mary's Hospital, has been appointed matron of the Military Hospital at Gibraltar. The War Office authorities are to be congratulated on the choice they have made for so important an appointment.

Miss MacIntyre has been appointed matron of the Carmarthenshire Infirmary in place of Miss Lambert, who resigned. Miss MacIntyre was trained at Westminster, and for many years sister of the Charlotte wards at the London Hospital.

Miss Frances Hale has been appointed matron of the National Orthopædic Hospital, in place of Miss Barnule. Miss Hale was lately assistant matron at Addenbrooke's Hospital, Cambridge.

Errata.—In last week's "Nursing Mirror," in the paragraph on the Zenana Mission, fourth line from the end, read "something" for "nothing."

### Vacancies.

The following vacancies are announced :—

**Matrons.**

Huddersfield Infirmary ; £80.

Cardiff Infirmary ; £80.

Dorset County Asylum ; £35.

**Nurses.**

Central London Ophthalmic Hospital; £35.

West Derby Union ; £25.

Barnhill Hospital, Glasgow ; £24.

County Hospital. Ayr ; £22 10s.

National Hospital, Queen-square ; £16.

Nursing Institution, Burton-on-Trent ; £25.

Swansea and South Wales Nursing Institute.

Burton-on-Trent Infirmary ; £25.

Ayr County Hospital ; £22 10s.

Weston-super-Mare Hospital ; £25.

Bolton Infirmary ; £25.

Nurses' Home, Truro.

Newark Hospital Nurses' Institute ; £20.

Birkdale Trained Nurses' Home.

Victoria Home, Bournemouth.

Royal Hospital for Diseases of Chest ; £22.

Brighton Borough Fever Hospital ; £25 12s.

Home Hospital, Fitzroy Square.

Hertford General Infirmary (assistant nurse) ; £16 to £20.

Children's Hospital, Sheffield.

**Midwife.**

Western Dispensary, Marylebone Road.

**Probationers.**

Brighton Borough Fever Hospital ; £18 12s.
Stratford-on-Avon Hospital.

